# Welcome to the Czech Republic again! Rare northern mosses *Calliergon megalophyllum* and *Drepanocladus sordidus* (Amblystegiaceae) in South Bohemia in light of their European distribution and habitat preferences

**DOI:** 10.3897/phytokeys.154.51454

**Published:** 2020-08-04

**Authors:** Łukasz Krajewski, Lubomír Adamec, Marta Saługa, Halina Bednarek-Ochyra, Vítězslav Plášek

**Affiliations:** 1 Institute of Technology and Life Sciences, Department of Nature Protection and Rural Landscape, 05-090 Raszyn, Poland Institute of Technology and Life Sciences Raszyn Poland; 2 Institute of Botany of the Czech Academy of Sciences, Department of Functional Ecology, 379 01 Třeboň, Czech Republic Institute of Botany of the Czech Academy of Sciences Třeboň Czech Republic; 3 W. Szafer Institute of Botany, Polish Academy of Sciences, 31-512 Kraków, Poland Polish Academy of Sciences Kraków Poland; 4 University of Ostrava, Department of Biology and Ecology, 710 00 Ostrava, Czech Republic University of Ostrava Ostrava Czech Republic

**Keywords:** Aquatic mosses, *Drepanocladus tenuinervis*, glacial relicts, *Hypnum moldavicum*, southern distribution, threatening, Třeboň Basin

## Abstract

Two aquatic moss species, *Calliergon megalophyllum* and *Drepanocladus sordidus* (Amblystegiaceae, Bryophyta), which had been considered extinct in the Czech Republic, were found in the Třeboň Basin, South Bohemia, in 2016–2017. They co-occurred in extensive reed- and sedge-dominated fen pools with humic water on the shore of an old fishpond and the former species was also discovered in a small humic pool in an old shallow sand-pit. The new Czech sites of these rare boreal species represent one of the southernmost known outposts within their entire European range. Previously, the two species were only known from single records in the Czech Republic from the late 19^th^ and early 20^th^ centuries. To confirm our morphological observations, we used phylogenetic analyses of DNA sequence variation in four chloroplast loci (*atp*B-*rbc*L, *trn*L-*trn*F, *rp*l16, *trn*G) and one nuclear region, the internal transcribed spacers of ribosomal DNA (ITS). We found (1) monophyly of all *Calliergon megalophyllum* specimens tested; (2) based on chloroplast DNA sequences, monophyly among all *Drepanocladus sordidus* specimens and representatives of *Pseudocalliergon turgescens* and *P. lycopodioides* moss species; (3) based on nuclear ITS sequences, monophyly of all original *D. sordidus* specimens. These results corroborate morphological studies and thus confirm the existence of natural sites for the studied moss species in the Třeboň Basin, South Bohemia, Czech Republic.

## Introduction

During a botanical excursion in August 2016 to the Ptačí blato fishpond in the Třeboň Basin (South Bohemia, Czech Republic), the lead author found two interesting aquatic moss species, *Calliergon megalophyllum* Mikut. and *Drepanocladus sordidus* (Müll. Hal.) Hedenäs. They have recently been considered extinct in the Czech Republic (category RE; Kučera et al. 2012). This site was revisited in 2017 and both species were found again, with *C. megalophyllum* additionally discovered in a small humic pool in an old shallow sand-pit at Branná in that area.

*Calliergon megalophyllum* was recorded for the first time in Central Bohemia and the specimen was described as a new species, *Hypnum moldavicum* Velen. (Velenovský 1903), but this has fallen into oblivion (cf. Hedenäs et al. 1999). This was apparently because Mikutowicz (1908) described *Calliergon megalophyllum* from Latvia, which gained wide acceptance in Scandinavian literature (Jensen 1939; Tuomikoski 1940; Nyholm 1965; Tuomikoski and Koponen 1979; Hedenäs 1993a, b, 1997) since this species is widely distributed there.

**Figure 1.**
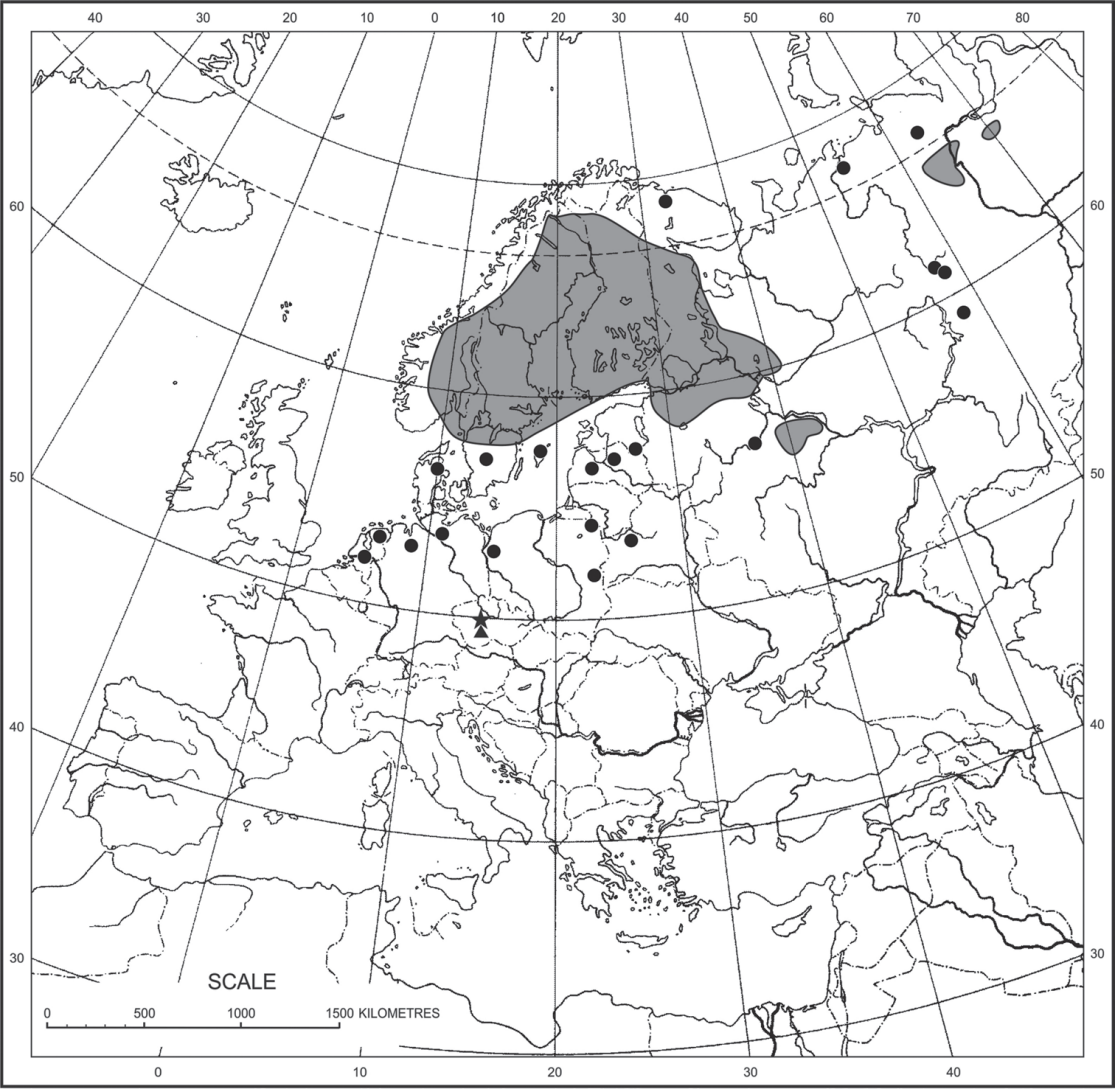
Distribution of *Calliergon megalophyllum* in Europe. The new locations in the Czech Republic are marked by the triangle and the extinct type locality of *Hypnum moldavicum* (cf. Velenovský 1903) is marked by the asterisk.

*Calliergon megalophyllum* is a panholarctic, subarctic-boreal species having a strongly dissected geographical range (Fig. 1). It has the main centre of its occurrence in Fennoscandia (Norway, Sweden, Finland), mainly within the latitudinal limits of approximately 60–70°N. Outside Fennoscandia, the species is widely distributed but scattered in the northern regions of European and Asian Russia (Afonina and Czernyadjeva 1995; Ignatov et al. 2006) and very rarely in the Northwest Territories, the Yukon and Alaska in North America (Hedenäs 2014a). In Europe, *C. megalophyllum* has occasionally been found at isolated sites at lower latitudes: in Russia, Estonia, Latvia, Poland, Germany, Denmark and The Netherlands (Karczmarz and Touw 1973; Hedenäs 2003a; Ignatov and Ignatova 2004; Meinunger and Schröder 2007; Kooijman et al. 2015), reaching its southernmost site in the Czech Republic (Velenovský 1903).

In the Czech Republic, *Calliergon megalophyllum* was collected only once in 1896 in Štĕchovice near Prague on the right bank of the Vltava river (German: Moldau) and described as *Hypnum moldavicum*. It has never been confirmed there since as this site was probably flooded by the dam reservoir and, accordingly, the species has been considered extinct (Váňa 2005a). Its Central European sites are relictual from the glacial period (Karczmarz 1971). Some of them no longer exist, for example in Germany, where it was reported at three sites in the northern part of the country (Meinunger and Schröder 2007). However, at least one German record is erroneous, because three specimens from Hagenmoor near Forst Klövensteen distributed by Bauer (1930) as No. 2092 in his *Musci Europaei et Americani Exsiccati* clearly represent *C. richardsonii* (Mitt.) Kindb. ex G. Roth (L. Hedenäs pers. com.). After many years, it has recently been rediscovered in The Netherlands (Kooijman et al. 2015). In Poland, several sites have been known in northern and eastern regions (Lisowski 1960; Ochyra and Tomaszewicz 1982; Ochyra and Szmajda 1983).

**Figure 2.**
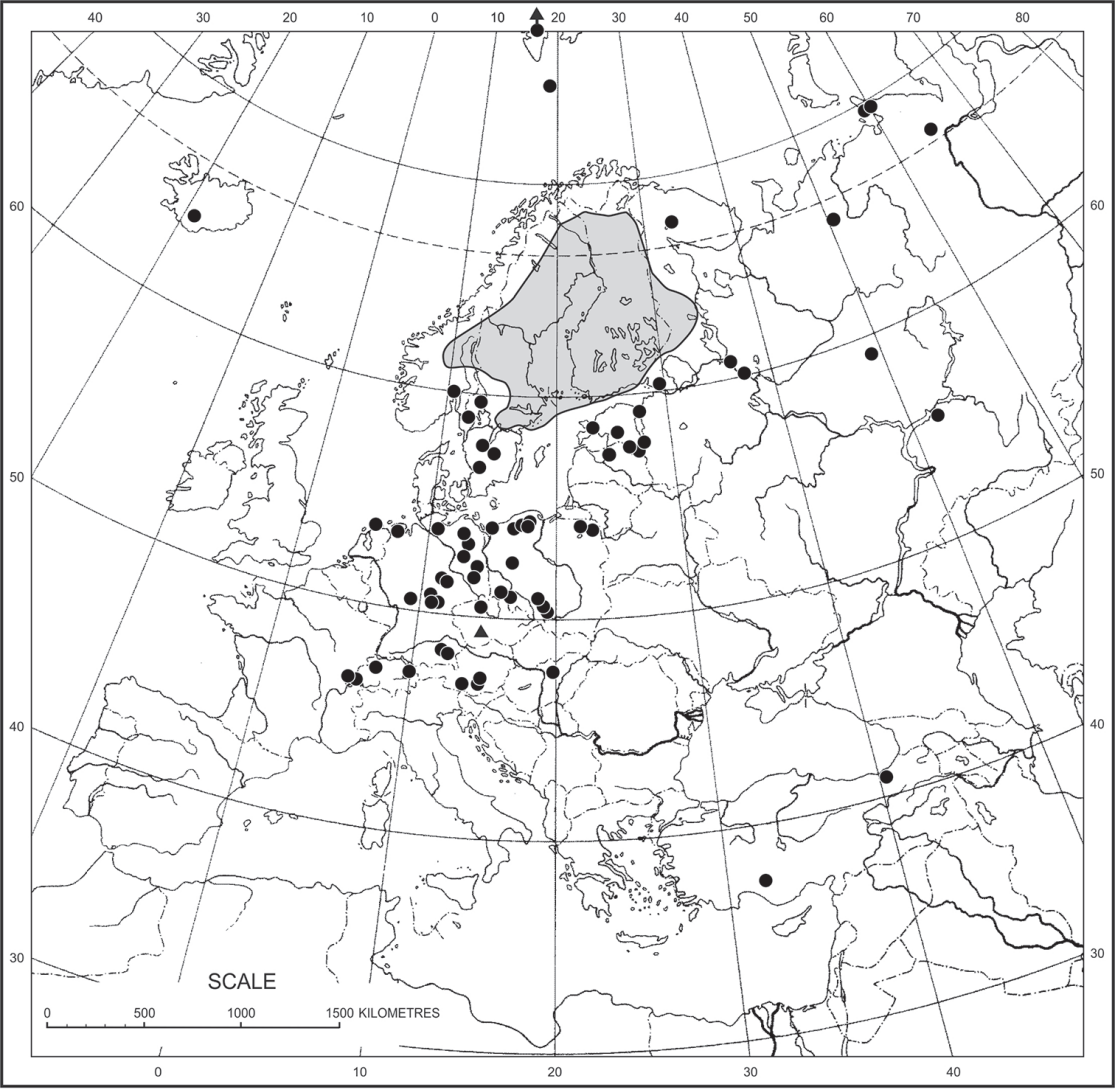
Distribution of *Drepanocladus sordidus* in Europe. The new locations in the Czech Republic are marked by the triangle. Occurrence on the northern coast of Spitsbergen beyond the map is indicated by the arrow.

*Drepanocladus sordidus* occurs in slightly mineral-rich to eutrophic habitats. It was recorded growing as a submerged or amphibious species in lakes, backwater pools and oxbows, terrestrial wetland habitats or fens. It is a panholarctic species having a strongly discontinuous geographical range in boreal and temperate zones (Fig. 2). It is most frequent in North America, ranging from Greenland, Nunavut and Alaska southwards to California, Oklahoma and Mexico (Hedenäs 2003b, 2014b), with some isolated sites in the West Indies and Central America and in the North and Central Andes in South America (Hedenäs 2003b). In Eurasia, it is mostly distributed in Sweden and Finland (Hedenäs 1993b) and very rarely in Iceland and Norway (Hedenäs 2003a), Karelia and in the Republic of Komi (Zheleznova 1994) and on Vaygach Island (Hedenäs, pers. com.) in north-eastern Europe, as well as in Taymyr Peninsula and Yakutia in Asia (Ignatov et al. 2006), extending as far north as Svalbard (Hedenäs 1998). In continental Europe, *D. sordidus* is very rare and scattered in the Netherlands (Hedenäs 1998), France (Hedenäs, pers. com.), Germany (Meinunger and Schröder 2007), Poland (Ochyra et al. 2003), Austria (Köckinger et al. 2008), Hungary (Erzberger and Papp 2004), Switzerland (Hedenäs and Bisang 2002), as well as Latvia (Āboliņa 2001) and Estonia (Ingerpuu et al. 1994). Additionally, *D. sordidus* was once recorded in the north-western part of the Czech Republic, in the Červený rybník fishpond near the village of Pihel close to the Česká Lípa town (Váňa 2005b). The moss was collected in 1910 by A. Schmidt and distributed by Bauer (1915) in his *Musci Europaei Exsiccati* as No. 1418 as D. lycopodioides (Brid.) Warnst. fo. immersus Mönk. The southernmost record of this species is in Turkey (Hedenäs 2003a). In general, *D. sordidus* has a somewhat wider range in Europe than *Calliergon megalophyllum* (Hedenäs 2003a), but the degree of threat to both species is similar (Hodgetts et al. 2019).

Although there are many known sites of the two aforementioned moss species, reliable data on biotic and abiotic habitat conditions are still scarce in the literature. In this paper, we characterise water chemistry and vegetation composition at both Czech sites. Also, we compare these characteristics with those at other European sites and discuss the phytogeographic particulars of both species in Europe. As the two species are rare and vanishing in the neighbouring countries (Hodgetts 2015), we briefly discuss the perspectives for their survival and their conservation concerns.

To investigate whether genetic data support species designation of individuals of *Calliergon megalophyllum* and *Drepanocladus sordidus* collected from the Třeboň Basin, Czech Republic, we sequenced the following DNA regions: nuclear ITS (internal transcribed spacers ITS1-5.8S-ITS2 of ribosomal DNA), and selected plastid DNA regions (intergenic spacer *atp*B-*rbc*L, intron of the *rpl*16 gene, intron of the tRNA^Gly^ (UCC) gene *trn*G, and *trn*L gene plus the adjacent *trn*L-*trn*F (GAA) spacer together as a single amplicon). In the first step, our original data was compared to the available nucleotide sequences of reported moss species found in the GenBank database. Secondly, apart from collected individuals, additional herbarium specimens of *C. megalophyllum* and *D. sordidus* from Poland and Finland were used as a part of the molecular research. Finally, the taxonomic status of described species was resolved using two methods: maximum likelihood (ML) and Bayesian inference (BI) employed for the dataset consisting of our original sequences supplemented by the GenBank resources. Hence, we placed described species into the larger context of Amblystegiaceae taxonomy of their closely related taxa, e.g. *Calliergon* (Sull.) Kindb., *Loeskypnum* H. K. G. Paul, *Straminergon* Hedenäs, *Warnstorfia* Loeske in the case of *C. megalophyllum*, and *Cratoneuropsis* (Broth.) M. Fleisch., *Drepanocladus* (Müll. Hal.) G. Roth, *Pseudocalliergon* (Limpr.) Loeske concerning *D. sordidus*. Lastly, we checked the morphological identification of these species with their clustering in phylogenetic trees based on nuclear and plastid DNA.

## Materials and methods

### Description of the mosses and the nomenclature

The nomenclature of mosses follows Kučera et al. (2012), algae Caisová and Gąbka (2009), vascular plants Danihelka et al. (2012), and syntaxonomy Chytrý (2011). The herbarium materials have been deposited in the Bryological Herbarium of the W. Szafer Institute of Botany of the Polish Academy of Sciences in Kraków (KRAM), University of Ostrava (OSTR), and Medical University of Silesia, Department of Pharmaceutical Sciences in Sosnowiec (SOSN).

### Description of the sites and field work

*Calliergon megalophyllum* and *Drepanocladus sordidus* were firstly found in the 1^st^ fen pool (as counted from the north) on the eastern shore of Ptačí blato fishpond, ca. 4 km west of Lomnice nad Lužnicí, Třeboň Basin Biosphere Reserve, South Bohemia, Czech Republic, in August 2016 (for details of the site, see Adamec 1999; Adamec and Lev 1999; Adamec and Kovářová 2006). The Ptačí blato fishpond is a hypertrophic fishpond with an irregular water inflow. It is regularly emptied at 2-yearly intervals in October and its normal water level is reached again in February-April. During the 2014–2019 seasons, it suffered from summer drought and its summer water level was usually 10–30 cm lower than expected. Its whole SE shoreline is adjacent to a partly afforested fen meadow. In the 1970s, ca. 12 shallow fen pools of 0.04–0.1 ha in size attached to the fishpond were excavated in the fen soil. Up to now, they have had various connections with the fishpond body and have been subject to various botanical successions (Adamec 1999; Adamec and Kovářová 2006). A rare aquatic carnivorous plant *Aldrovanda vesiculosa* L. (Droseraceae) was successfully introduced to some pools in 1995 and a stable and abundant population has arisen there (Adamec and Lev 1999; Adamec and Kovářová 2006). On 28 June 2017, a detailed search for both moss species was conducted in the first 10 fen pools. The water level in the dominant parts of the moss stands was <6 cm.

On 12 October 2017, *Calliergon megalophyllum* was also found in a small shallow humic pool (area ca. 70 m^2^, depth 3–50 cm) in an old sand-pit complex near Branná, ca. 4 km SE from Třeboň (Adamec 2009, 2010; Adamec and Kučerová 2013). To characterise the microsites for either of the rare moss species, we conducted a floristic survey (vegetation dominants) and measured basic parameters of water chemistry (pH, electrical conductivity) in typical microhabitats of both species by portable meters.

In July 2017, *Calliergon megalophyllum* was searched for comparison in NE Poland, in three historically known sites in Suwałki Landscape Park (cf. Ochyra and Tomaszewicz 1982), but was found only at one site near Błaskowizna on 17 July 2017. A very wet mire on thick peat deposits is developed there in a depression, surrounded by parallel, arched and prominent ridges of moraines (glacial curvilineations created by catastrophic megafloods during last glaciation; Weckwerth et al. 2019). The vegetation represents *Pinus*-*Betula* swamp forests, peatbogs and fens, with many relic species (most notable: *Eriophorum gracile* W. D. J. Koch ex Roth, *Baeothryon alpinum* T. V. Egorova, *Pseudocalliergon trifarium* (F. Weber & D. Mohr) Loeske and *Scorpidium scorpioides* (Hedw.) Limpr.) occurring, but *C. megalophyllum* was found there in an old peat hollow. Subsequently, water chemistry measurements were repeated on 27^th^ July 2017 (Elmetron CPC-401).

### Other potential sites of the two moss species checked in the Třeboň Basin

To specify the occurrence of both rare moss species at potential, humic mesotrophic sites within the Třeboň Basin, an extensive search for both species was conducted at 44 sites in this region in the 2017 and 2018 seasons (see the list in the Suppl. material 1). The potential sites were selected based on similarity with those (micro)sites of both mosses at Ptačí blato and Branná according to the following criteria: shallow standing humic water, loose reed- or sedge-dominated wetland vegetation, highly organic bottom sediment consisting of reed or sedge litter or fen, partial shading by adjacent vegetation, proximity to eutrophic fishponds but evidently low levels of nutrients (N and P). As both rare moss species were found at both Czech sites where aquatic carnivorous plants also grow (both naturally spread and introduced; see Adamec and Kučerová 2013), it is possible to suggest that the ecological requirements for the rare mosses and aquatic carnivorous plants *Aldrovanda vesiculosa*, *Utricularia australis* R. Br., *U. bremii* Heer, *U. ochroleuca* R.W. Hartm. *s. str.* greatly overlap. Therefore, the potential sites were also selected according to the occurrence of any of these aquatic carnivorous species. Moreover, in the past, many of these sites were used for ecophysiological studies on this plant group and some data on water chemistry are available (Adamec 1999, 2008, 2009, 2010; Adamec and Lev 1999; Adamec and Kovářová 2006). The distance between any of these selected sites and those of the rare mosses never extended beyond 20 km.

### Determination of the mosses

*Calliergon megalophyllum* is a stenotypic species which exhibits a narrow range of morphological variability and is easily distinguishable from other congeners. It is usually a very robust moss, usually growing submerged and often floating, in somewhat nutrient-rich lakes, oxbow lakes and other small water bodies. The plants are green, brownish or yellowish, with shoots 15–30 cm long (exceptionally to almost 1 m in Sweden, L. Hedenäs pers. com.), radially, slightly pinnately or irregularly branched. The stem leaves are erect-spreading, concave, broadly rounded or rounded-ovate to rounded ovate-cordate, 3.5–5.0(–6.0) mm long, 2.5–3.0(–4.5) mm wide, broadly rounded at the apex and abruptly narrowed into a short blunt point. The costa is single, unbranched, 40–80(–105) µm wide at the base, ceasing just below the leaf apex. The alar cells are large, hyaline and thin-walled, forming a large pellucid group sharply separated from the adjacent laminal cells and occupying nearly two third or less the leaf base. *Calliergon megalophyllum* is closely related to *C. richardsonii*, but the latter differs in its shorter costa, ending far below the apex which is branched or spurred below and usually forked at the apex. The costa of *C. megalophyllum* is clearly thinner than that of *C. giganteum*, in which it is strong or very strong, 90–280 μm near the base, but it is also unbranched and extends to the leaf apex. The alar cells of *Calliergon cordifolium* (Hedw.) Kindb. form also a large group, reaching almost the costa but it is diffusely delimited and they transform gradually into laminal cells. Finally, a good differentiating character between *C. megalophyllum* and *C. richardsonii* is found in the relative length of the distal cell of the axillary hairs (more elongate in *C. richardsonii*). Since such hairs are abundant and large in *Calliergon* s.str., this is a useful character, especially when the identity of specimens is doubtful (L. Hedenäs pers. com.).

*Drepanocladus sordidus* belongs to the *D. sendtneri*-group species characterised by the presence of rounded-triangular groups of alar cells reaching from the leaf margin one third to two thirds of the distance to the costa. This character immediately distinguishes it from the *D. aduncus* group in which the alar cells form much larger, triangular groups reaching the costa. *Drepanocladus sordidus* is closely related to *D. sendtneri*, but it has a relatively weak costa, 30–75 µm wide near the base which vanishes far below the leaf apex and thin-walled alar cells. In contrast, *D. sendtneri* has a strong costa, (50-)70–100 µm wide near the base, ending in the leaf acumen near the apex and the alar cells have mostly incrassate walls. According to Hedenäs (2003a) the crucial difference between the two species is the ratio of the length of leaf cells (μm) to leaf length (mm). In *D. sordidus* this ratio is 23.3–36.5, whilst in *D. sendtneri* it is 17.9–24.4. The two species differ also in their ecological requirements. *Drepanocladus sordidus* prefers less mineralised waters (electrical conductivity only 0.1–2.4 mS/m, n = 45), whereas *D. sendtneri* prefers much harder waters (conductivity 14.0–96.0 mS/m, n = 19) (Hedenäs 1998, 2003a). Thus, the former usually occurs in oligotrophic and dystrophic habitats (e.g., *Lobelia* lakes), while the latter grows typically in alkaline fens (Rydin et al. 1999).

### Preparation of outdoor culture

To keep living material of the two exceedingly rare moss species available for any study purpose, a simple outdoor culture of them originating from 2–3 specimens collected from Ptačí blato (first pool) has been established in the collection of aquatic and wetland plants in the Institute of Botany CAS at Třeboň (CZ 0 HBT 2017.03851; CZ 0 HBT 2017.03802). The cultivation mimicked the natural conditions of both species in humic water and was the same as that used for growing aquatic temperate *Utricularia* species (e.g., Sirová et al. 2003). Each moss species was grown in a 3 litres shaded aquarium floating in an outdoor 1 m^2^ plastic container for cooling. A litter of robust sedges and a small amount of fen soil were used as a substrate which rendered the water humic (pH was ca. 6.6–7.0). Both moss species have grown vigorously in this culture and have formed a substantial biomass, but older parts of their shoots were covered by filamentous algae.

### Materials, DNA extraction, PCR amplification and DNA sequencing

One sample of *Calliergon megalophyllum* and *Drepanocladus sordidus* was collected in the field (Třeboň Basin, Czech Republic), with voucher specimens deposited in the Bryophyte Herbarium at the W. Szafer Institute of Botany of the Polish Academy of Sciences (KRAM) under the following vouchers: *C. megalophyllum* (KRAM B-249804), and *D. sordidus* (KRAM B-249802). Tissue samples of the above mentioned specimens in the form of leafy gametophyte fragments were stored in silica gel until genetic analysis was performed. The sampling was completed using four herbarium specimens from the KRAM collection: two individuals of *C. megalophyllum* (Poland, KRAM B-177615 and KRAM B-249803) and two individuals of *D. sordidus* (Finland, KRAM B-55364; Poland, KRAM B-75950). All specimens were morphologically verified before genetic analysis.

Gametophyte fragments of weight equal to 12 mg of both herbarium and fresh samples were used for DNA extraction. Total genomic DNA was isolated from six individuals with the Isolate II Plant DNA Kit (Bioline, Meridian Life Science, Memphis, USA) following the manufacturer’s guidelines. We amplified and sequenced five DNA regions that were previously used to analyse the phylogenetic relationships in these genera. Accordingly, the internal transcribed spacers (ITS1-5.8S-ITS2) of nuclear DNA, and the following plastid regions: *atp*B-*rbc*L, *trn*L-*trn*F, *trn*G, and *rpl*16 were used on three individuals of *C. megalophyllum*. The internal transcribed spacers (ITS1-5.8S-ITS2) of nuclear DNA, and plastid *atp*B-*rbc*L, *trn*L-*trn*F regions were tested on three individuals of *D. sordidus*. The analysed DNA fragments were amplified with primers according to PCR conditions described in Saługa et al. (2018). Negative controls were added to all PCR reactions. PCR products were sequenced in both directions with an AB3500 sequencer. Resulting chromatograms were edited and contigs were assembled using Geneious v.10.1.3 (Biomatters Ltd.). All nucleotide sequences reported in this study have been deposited in GenBank with accession numbers given in Suppl. material 3. DNA extracts are deposited in the Laboratory of Molecular Analyses at the W. Szafer Institute of Botany of the Polish Academy of Sciences.

### Phylogenetic analyses

To evaluate the genetic designation of described species we combined the sequences newly obtained in this study with the previously published sequences collected from BLAST searches of the GenBank database. In the first step, our original sequences were compared to molecular data of *Calliergon megalophyllum* (Sweden, MAC B88612), and *Drepanocladus sordidus* (USA, S B39576) specimens. Afterwards, selected molecular data published by Hedenäs et al. (2005) and Hedenäs (2006) was used to construct *C. megalophyllum* phylogeny. Original DNA sequences generated from *D. sordidus* specimens were analysed along with selected sequences published by Hedenäs and Rosborg (2008), and Saługa et al. (2018) (see Suppl. material 3). Sequence alignments were conducted in Geneious v.10.1.3 (Biomatters Ltd.) using the Geneious Alignment option. Final editing was done using BioEdit Sequence Alignment Editor v7.2.5 (Hall 1999). For each of the alignments, the programme jModelTest 2.0 v.0.1.1 (Guindon and Gascuel 2003; Darriba et al. 2012) was used to determine the model of sequence evolution. For the *C. megalophyllum* phylogeny, model selection using the Akaike Information Criterion (AIC), resulted in the GTR+I+G (ITS), TPM1uf+I (*atp*B-*rbc*L, *rpl*16), TPM3uf+I (*trn*G), and HKY+I (*trn*L-*trn*F) models of sequence evolution. The following evolutionary models were selected for the *D. sordidus* phylogeny: HKY+G (ITS), TPM1uf (*atp*B-*rbc*L), and TIM1+G (*trn*L-*trn*F). For both species, nuclear and plastid sequences were analysed separately, except that all plastid sequences were combined into a single data matrix. The SeaView v.4 (Gouy et al. 2009) was used to concatenate all analysed plastid sequences. To infer phylogenetic trees, we applied two approaches: Bayesian inference (BI) in MrBayes v.3.2.6 (Ronquist et al. 2012) and Maximum Likelihood (ML) in RAxML v.7.2.4 (Stamatakis 2006; Stamatakis et al. 2008). One thousand non-parametric rapid bootstrap replications were used to generate ML trees. All ML analyses performed for *C. megalophyllum* phylogeny were conducted using the GTR GAMMAI model of sequence evolution (GTR + I + G). This is due to the fact that Stamatakis (2006) has only implemented the proportion of invariable sites parameter (I) along with gamma distributed variable sites (G) in RAxML. With regard to *D. sordidus* analyses, we used the GTR GAMMA sequence evolution model. As a starting tree for full ML searches, every fifth bootstrap tree was used. The resulting trees with the highest ML scores were chosen.

In the BI analyses, each target plastid region was treated as a separate partition during the analyses. Here, two independent runs starting from random trees were applied, each using four Markov chains. All analyses were run for 10,000,000 generations with sampling trees every 100 generations. In the final analysis 25 per cent, burn-in trees were discarded and the remaining trees and their associated parameter values were saved. The convergence of the chains was determined by examining the plot of all parameters values and the -lnL against generation time using the programme Tracer v.1.5, as recommended by Drummond and Rambaut (2007) to analyse MrBayes and BEAST output files.

## Results

### Distribution

A list of (micro)sites where the mosses were recently recorded.

*Calliergon megalophyllum*

Czech Republic:

1a) 3.5 km WNW of Lomnice nad Lužnicí town, Ptačí blato fishpond, NE part, loc. Zátoka 1, 49°5.45'N, 14°40.183'E, and nearby fen meadow, 49°5.4184'N, 14°40.2793'E, 434 m a.s.l., leg. Ł. Krajewski, 20 Aug 2016 (KRAM B-249804), leg. R. Ochyra with V. Plášek, H. Bednarek-Ochyra, Ł. Krajewski and L. Adamec, 28 June 2017 (KRAM B-250940, B-250945, B-250946, SOSN 67668).

1b) 3.5 km WNW of Lomnice nad Lužnicí town, Ptačí blato fishpond, NE part and nearby fen meadow, loc. Zátoka 2, 49°5.4106'N, 14°40.2562'E, 434 m a.s.l., leg. R. Ochyra with V. Plášek, H. Bednarek-Ochyra, Ł. Krajewski and L. Adamec, 28 June 2017 (KRAM B-250948).

1c) 3.5 km WNW of Lomnice nad Lužnicí town, Ptačí blato fishpond, NE part and nearby fen meadow, loc. Zátoka 4, 49°5.3873'N, 14°40.231'E, 434 m a.s.l., leg. R. Ochyra with V. Plášek, H. Bednarek-Ochyra, Ł. Krajewski and L. Adamec, 28 June 2017 (KRAM B-250950).

2) 4 km SE of Třeboň town, sand-pit near Branná village, small humic pool in the complex on the margin of a forest, 48°58.428'N, 14°47.813'E, 440 m a.s.l., leg. V. Plášek, 12 Oct 2017 (OSTR B-7253, KRAM B-253888).

Poland:

3) 1 km NE of Błaskowizna, mire E of Boczniel Lake (E of Hańcza Lake), hollow in peat, 54°15.458'N, 22°49.433'E, 230 m a.s.l., leg. Ł. Krajewski, 17 Jul 2017 (KRAM B-249803, SOSN 67667).

*Drepanocladus sordidus*

Czech Republic:

1a) 3.5 km WNW of Lomnice nad Lužnicí town, Ptačí blato fishpond, NE part, loc. Zátoka 1, 49°5.45'N, 14°40.183'E, 434 m a.s.l., leg. Ł. Krajewski, 20 Aug 2016 (KRAM B-249802), leg. R. Ochyra with V. Plášek, H. Bednarek-Ochyra, Ł. Krajewski and L. Adamec, 28 June 2017 (KRAM B-250941, B-250942, B-250943, B-250944, B-250947).

1b) 3.5 km WNW of Lomnice nad Lužnicí town, Ptačí blato fishpond, loc. Zátoka 3, 49°5.3993'N, 14°40.183'E, 434 m a.s.l., leg. R. Ochyra with V. Plášek, H. Bednarek-Ochyra, Ł. Krajewski and L. Adamec, 28 June 2017 (KRAM B-250949).

Specimens of *Calliergon megalophyllum* recorded in Ptačí blato fen pools in 2016 were relatively small (shoots 10–20 cm long, leaves up to 4 mm long and 3.5 mm wide) overgrown with periphyton, dark green-brown, with leaves protruding at right angles and distant, just like C. megalophyllum var. natans Karczm. (Karczmarz 1971; Karczmarz and Touw 1973), but branching out. Despite the smaller size, the shape, the strongly concave leaves, the size and location of the alar cell groups were typical for the species. In 2017, the species grew in much shallower water, occasionally almost as a terrestrial plant.

Specimens of *Drepanocladus sordidus* from Ptačí blato pools in 2016 were strongly overgrown with periphyton, usually dark and dying in the opaque, humic water. The alar cells were typical of the species, the habit turned out (10–20 cm), the leaves strongly bent. Thus, the co-occurred form of *D. aduncus* (Hedw.) Warnst. growing in the pools is macroscopically distinguishable in much less curved leaves.

In the first (northernmost) pool, both *Calliergon megalophyllum* and *Drepanocladus sordidus* were found in a scattered stand on an area of >200 m^2^. The shallow water in the pool is strongly coloured with humic acids, which is partly caused by the adjacent fen meadow. Yet the water chemistry in the pool is also influenced by the nutrient-rich, hard water from the fishpond body, the penetration of which depends on water level (Adamec and Lev 1999). Vegetation within the microsite is represented by the weakly compact association *Typhetum angustifoliae* Pignatti 1953, where the dominant stands are formed by *Typha angustifolia* L., *Phragmites australis* (Cav.) Trin ex Steud., *Carex rostrata* Stokes, *C. paniculata* L., *Juncus effusus* L. and J. bulbosus var. fluitans (Lam.) Beck. Also the macrophytes associated with nutrient-poor, low-mineralised waters were observed there, e.g. *Utricularia australis* R. Br., *Riccia fluitans* L., *Nitella gracilis* (Sm.) Ag., *N. flexilis* (L.) Ag., *Chara delicatula* Ag. and *Chara globularis* Thuill.

Only isolated shoots of *Calliergon megalophyllum* were found interspersed on the eastern fen margin of the first pool adjacent to an afforested fen meadow within *Carici elatae* – *Calamagrostietum canescentis* Jílek, 1958 (variant with *Comarum palustre* L.). They grew in the vegetation dominated by *Calamagrostis canescens* (Weber) Roth, *Agrostis canina* L., *Juncus effusus*, *Peucedanum palustre* (L.) Moench, *Lycopus europaeus* L., *Viola palustris* L., *Hydrocotyle vulgaris* L. and *Sphagnum* spp.

Another site of *Calliergon megalophyllum* was also found in the Třeboň Basin - in a small humic pool in the sand-pit complex near Branná. The very dense and dominant stand occupies an area of >30 m^2^, which amounts to ca. half of the surface area of the pool. Although the total water column in the moss stand is 20–40 cm deep, the water is completely filled by plant dominants (*Comarum palustre* L., *Calliergon megalophyllum*, *Hydrocharis morsus-ranae* L.) and the moss grows only near the water surface and an apical part of the shoots often emerges. At this site, the moss also grew scarcely along the adjacent shoreline of a large shallow sand-pit pool in a ca. 20 m long reach. It was evident that these plants were dispersed from the small pool during the events of high-water level or transferred by large animals.

In summary, both moss species grew in relatively soft waters (conductivity 8.8–19.8 mS/m) and within a narrow pH range of 5.94–7.04 in the Třeboň Basin and NE Poland (Table 1), which is in agreement with data from other European sites (Suppl. material 1).

**Table 1.** Summary of vegetation and abiotic factors found at microsites of *Calliergon megalophyllum* (CM) and *Drepanocladus sordidus* (DS) at CZ: Ptačí blato and Branná sites, Třeboň Basin Biosphere Reserve, South Bohemia, Czech Republic, in 2017 and PL: Błaskowizna, Suwałki Landscape Park, NE Poland. The pools at Ptačí blato are counted from the north.

No. of the pool	Water depth (cm)	Plant dominants	Rare mosses	Electr. conduct. (mS/m)	pH	Comments on rare moss abundance
a) CZ: Fen pools at Ptačí blato fishpond						
1^st^	0–5	*Phragmites australis*, *Carex* spp., *Typha angustifolia*, *Juncus effusus*, *Utricularia australis*, *Lythrum salicaria*	CM, DS	13.5–19.5	6.13–6.42	both species common
4^th^	0–4	*Carex* spp., *Sparganium erectum*, *Utricularia ochroleuca*, *Agrostis canina*	CM	13.8–15.8	6.24–6.36	CM scarcely
9^th^	0	*Calamagrostis canescens*, *Juncus effusus*, *Galium uliginosum*, *Carex rostrata*, *Lysimachia vulgaris*	DS	–	–	DS very scarcely
b) CZ: Fen meadow E of 1^st^ bay of Ptačí blato fishpond						
	0–5	*Calamagrostis canescens*, *Juncus effusus*, *Hydrocotyle vulgaris*, *Viola palustris*, *Sphagnum* spp.	CM	15.8	6.36	CM very rare
c) CZ: Humic pool in Branná sand-pit						
Open stand	20–40	*Comarum palustre*, *Hydrocharis morsus-ranae*	CM	11.2	6.04–6.08	dominant
Shaded stand	20–30	*Comarum palustre*, *Carex acuta*, *Lysimachia vulgaris*	CM	8.8	5.94–6.10	dominant
d) PL: Peat hollow in mire E of Boczniel Lake						
	70–150 (floating)	*Comarum palustre*, *Myriophyllum verticillatum*, *Hydrocharis morsus-ranae*, *Sparganium natans*, *Stratiotes aloides*, *Calliergon giganteum*, *Utricularia* spp.	CM	16.9–19.8	6.95–7.04	CM rare

### Molecular recognition of *Calliergon megalophyllum* and *Drepanocladus sordidus*

Sequence alignments of selected target DNA regions support the recognition of two individuals of aquatic moss species in the study area: *Calliergon megalophyllum* and *Drepanocladus sordidus*. Our original sequences obtained from the examined specimens are homogeneous in all DNA regions tested. The only minor detected genetic differences, if present, concern original versus GenBank DNA sequences.

Genetic analysis of *Calliergon megalophyllum* specimens resulted in the uniform plastid and nuclear data within the testing group (Czech Republic, KRAM B-249804; Poland, KRAM B-249803 and KRAM B-177615; Sweden, MAC B88612) with two single nucleotide polymorphisms (SNP’s) detected only from *trn*L-*trn*F plastid region. Recognised SNPs are related to the sequence ends, which could come up as an effect of the technical sequencing errors.

Similar results can be found by comparing plastid DNA sequences within the group of *Drepanocladus sordidus* specimens (Czech Republic, KRAM B-249802; Poland, KRAM B-75950; Finland, KRAM B-55364; USA, S B39576). All sequences are identical, with only three SNPs detected from *trn*L-*trn*F intergenic spacer, likewise at the ends of the sequences. Quite opposite, in the group of nuclear ITS sequences of *D. sordidus* specimens we found, four SNPs, and two insertion/deletion polymorphisms (indels), which differ in the original sequences generated with this study from data in GenBank.

### Bayesian and maximum likelihood species delimitation

**Figure 3.**
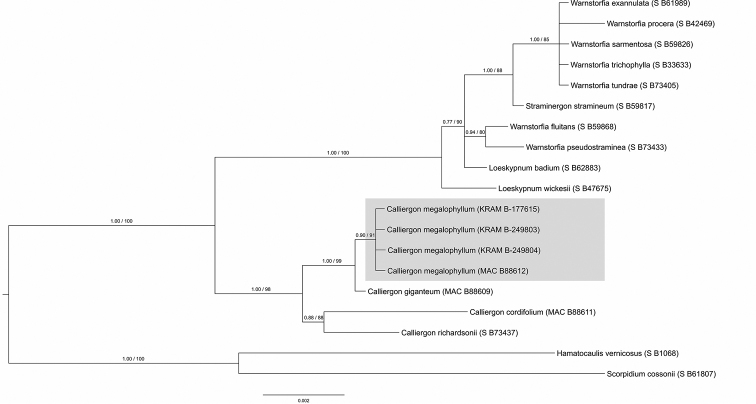
Cmega_concatenated plastid BI and ML analysis.

**Figure 4.**
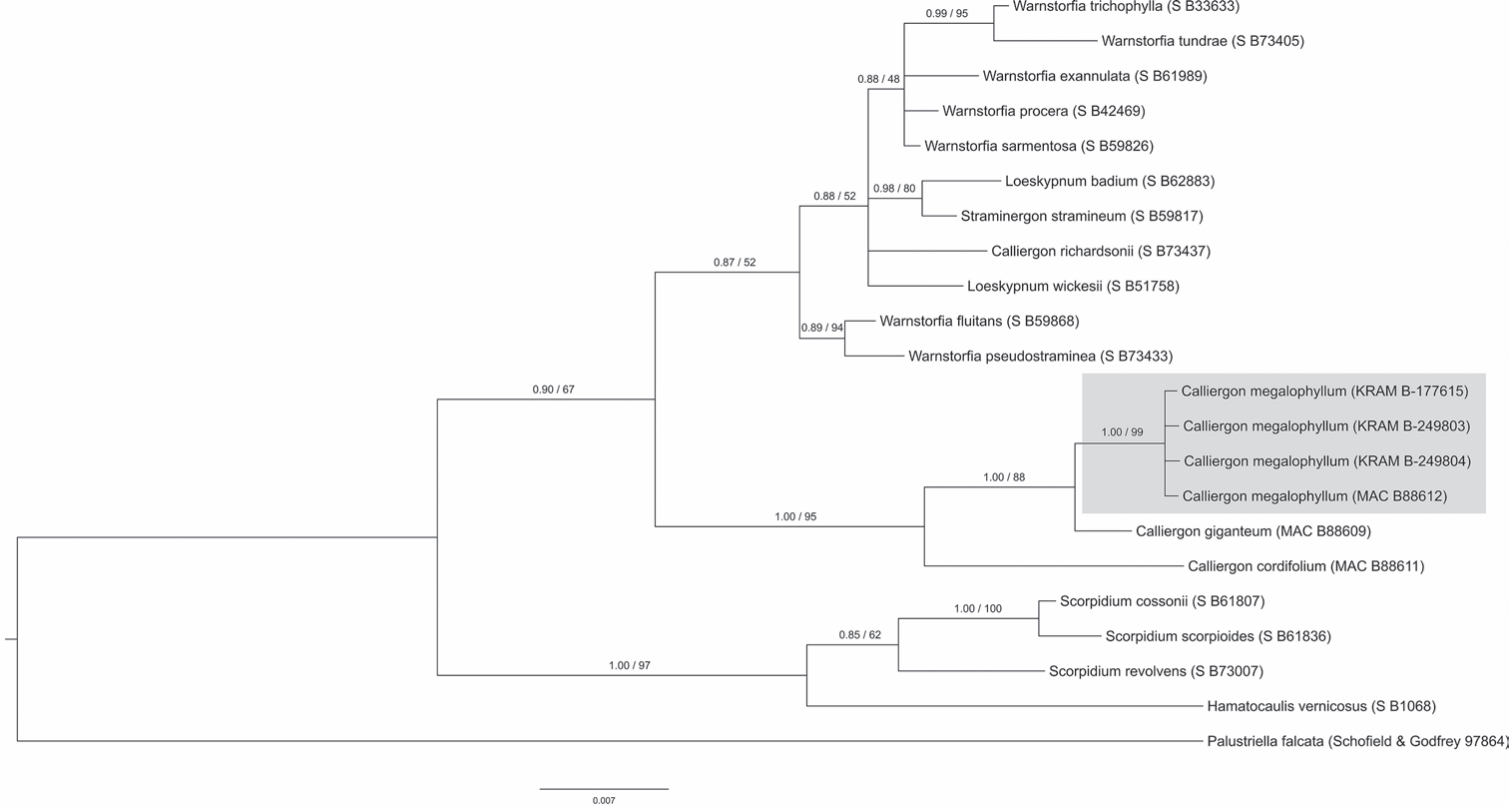
Cmega_ITS BI and ML analysis.

**Figure 5.**
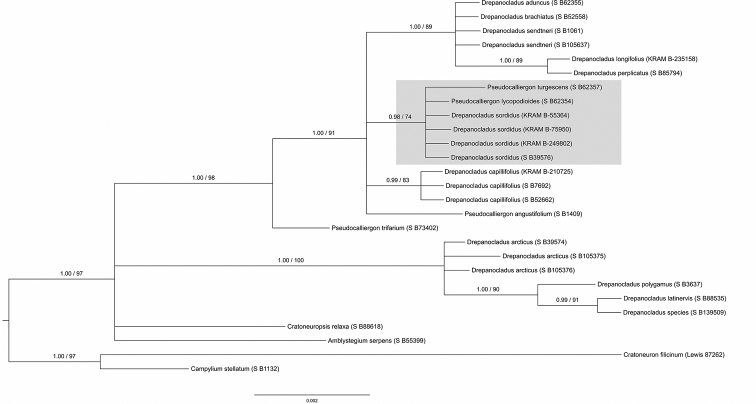
Dsor_concatenated plastid BI and ML analysis.

**Figure 6.**
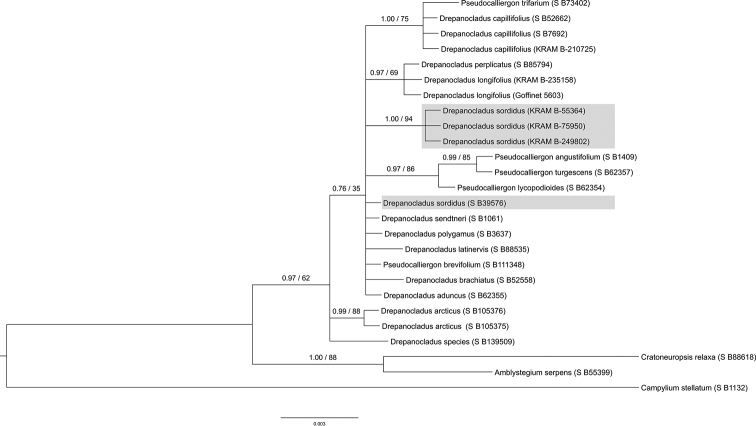
Dsor_ITS BI and ML analysis.

Species recognition of the specimens of *Calliergon megalophyllum* and *Drepanocladus sordidus* collected from the Třeboň Basin was well supported by all BI and ML analyses (see Figs 3, 4, 5, 6). Both of the guide BI and ML trees for either the nuclear (ITS) or selected plastid data (*atp*B-*rbc*L, *rpl*16, *trn*G, *trn*L-*trn*F) represent identical topologies, with posterior probabilities (PP), and bootstrap support (BS) values of greater than or equal to 0.90 (PP), and 90% (BS), calculated for the split between the studied species and their closest relatives. The only exception is the plastid based ML tree obtained for *D. sordidus* moss species, for which bootstrap support is lower than 90%, and is equal to 74%. Both support values (PP; BS) are presented at single, representative nuclear ITS or plastid trees for a given moss species.

The plastid and nuclear ITS analyses of *Calliergon*, *Loeskypnum*, *Straminergon*, and *Warnstorfia* produced very similar tree topologies, and both have resulted in a monophyletic, well-supported clade consisting strictly of *C. megalophyllum* specimens represented by the following individuals: Czech Republic, KRAM B-249804; Poland, KRAM B-249803 and KRAM B-177615; Sweden, MAC B88612, suggesting that they are genetically homogeneous. In contrast with the above, phylogenetic analyses of *Drepanocladus*, *Cratoneuropsis*, and *Pseudocalliergon* inferred from plastid and nuclear ITS data resulted in different tree topologies. Based on the concatenated plastid sequences it can be concluded that all representatives of *Drepanocladus sordidus* are genetically uniform (Czech Republic, KRAM B-249802; Poland, KRAM B-75950; Finland, KRAM B-55364; USA, S B39576). However, representatives of *Pseudocalliergon lycopodioides* (Brid.) Hedenäs, and *P. turgescens* (T. Jensen) Loeske are clustering together with the abovementioned specimens, and all form a monophyletic, well-supported clade with Bayesian posterior probabilities (PP) but on the other hand they were not strongly supported with bootstrap resampling values (BS). Accordingly, we tested, in pairwise comparisons, the sequence identity within the described clade. Apart from minor genetic incompatibility detected among *D. sordidus* representatives (see section "Molecular recognition of *Calliergon megalophyllum* and *Drepanocladus sordidus*"), we found only one SNP mutation in *atp*B-*rbc*L intergenic spacer with regard to *P. turgescens* specimen.

In the nuclear ITS trees our original *Drepanocladus sordidus* specimens (Czech Republic, KRAM B-249802; Poland, KRAM B-75950; Finland, KRAM B-55364) are recognised as a monophyletic clade with high (PP) and (BS) support values. Although this clade comprised all three analysed individuals, it does not include *D. sordidus* specimen (USA, S B39576) whose sequence was downloaded from GenBank data base. Although our original ITS sequences of *D. sordidus* specimens are well differentiated (four SNP’s and two indels) from the sequence of *D. sordidus* individual (USA, S B39576), their potential relationship is not well resolved. In the ITS tree, accession of *D. sordidus* (USA, S B39576) is placed in the ambiguous position among four monophyletic clades along with *D. aduncus*, *D. brachiatus* (Mitt.) Dixon, *D. latinervis* Warnst., *D. polygamus* (Schimp.) Hedenäs, *D. sendtneri* (Schimp.) Warnst., and *Pseudocalliergon brevifolium* (Lindb.) Hedenäs representatives. Here, contrary to plastid data, *P. turgescens* and *P. lycopodioides* together with the accession of *P. angustifolium* Hedenäs form a separate well-supported clade.

## Discussion

Ptačí blato fishpond (about 40 ha) was built at the end of the 16th century and represents one of the ca. 500 fishponds in this basin (Jeník and Květ 2002; Jeník et al. 2002). In 1872–1881, the pond was drained and the peaty soil from the bottom was extracted for fuel (Anonymus 2019). This indicates that at that time, some fen pools filled with humic water existed on the E shoreline of the fishpond, so rare mosses could survive there and a previously large mire was developing there for many centuries. During the 1970s, the fishpond managers excavated *ca.* 12 shallow pools (bays, lagoons) in the fen soil attached to the fishpond along its 400 m SE shoreline. The pools (of different sizes) have changed their connection with the body of the increasingly eutrophic fishpond since. Accordingly, they have been subject to variable botanical succession or even infilling by organic material and eutrophication (*cf.*Adamec 1999; Adamec and Lev 1999; Adamec and Kovářová 2006). The underlying rapid botanical succession has even accelerated due to the very dry 2014–2019 seasons. In contrast to the conditions in 1994–1995, when the dominant stands in several pools were formed by loose mesotrophic stands dominated by *Phragmites australis* and *Carex rostrata*, recent extensive helophyte stands in various pools are dominated by dense stands of mainly *P. australis*, *Typha angustifolia*, *Carex* spp. and *Juncus effusus* (Adamec, unpubl. obs.). In the first pool, both rare mosses co-occur with *Aldrovanda vesiculosa* which has been successfully introduced (Adamec and Lev 1999).

Presumably, *Calliergon megalophyllum* and *Drepanocladus sordidus* previously occurred in the area of the recent Ptačí blato fishpond (before the fishpond was built) in shallow, temporary inundations in *Caricion nigrae* fens, developed on peats (later mostly exploited and inundated), similar to e.g. recent *C. megalophyllum* sites in The Netherlands (Kooijman et al. 2015). This relic occurrence of *C. megalophyllum* could still be observed east of the 1^st^ pool, where single shoots survived in the natural fen vegetation. After the creation of the fishpond, both species dispersed and have become abundant in nearby, anthropogenic habitats of fishpond pools (e.g. by zoochory of wild boars). This was observed for *Pseudocalliergon turgescens* and other rare, northern-distributed mosses in southern Poland, which occurred there in large sand-pits, but almost disappeared in nearby fens (Krajewski 2017).

Also, the other Czech site of *Calliergon megalophyllum*, a sand-pit near Branná, is a typical man-made wetland in which sand extraction ceased perhaps in the 1950s. The small humic pool inhabited by the moss species is partly surrounded by a mixed forest so that falling leaves and branches permanently increase the trophic status to recent meso-eutrophy, and render the water strongly humic (Adamec 2008, 2009, 2010; Adamec and Kučerová 2013). Considering the recent occurrence of both rare mosses in S Bohemia, it is necessary to add that both species are, in fact, sterile and therefore, they can spread only as shoot fragments on the body of large animals (e.g., water birds, roe deer) probably for shorter distances, but there is known to be a dispersy even by small birds, migrating to long distances (Lewis et al. 2014).

The Třeboň Basin in the Czech Republic is a refuge for many glacial relics both of vascular plants and bryophytes, e.g. *Eriophorum gracile* (Kaplan et al. 2015), *Helodium blandowii* (F. Weber et D. Mohr) Warnst., *Meesia triquetra* (Jolycl.) Ångstr. (Holá et al. 2010). Many of them were also documented in the Třeboň Basin as macrofossils in Late-Glacial and Early-Holocene peat deposits (Hájková et al. 2018). Ptačí blato fishpond is located within the Třeboň Biosphere Reserve and Protected Landscape Area. However, a strong impact of the hypertrophic fishpond since the 1980s (liming, fertilisation by organic compost, overstocking by fishstock; see Pechar et al. 2002) on the adjacent fen meadow and excavated fen pools has caused a gradual deterioration of water chemistry factors towards eutrophication and reduction of water transparency (*cf.*Adamec 1999; Adamec and Lev 1999; Adamec and Kovářová 2006). This trend towards eutrophication is greatly amplified by very low water level in the fishpond and adjacent pools due to dry summer seasons since 2014, which has resulted in rapid overgrowth of the pools by dense robust helophyte stands. However, studies from Finland indicate that *Calliergon megalophyllum* is rather resistant to moderate eutrophication and could increase its abundance and even become dominant in the small bays of larger lakes or in small water bodies (Rintanen 1996).

The historic north-west Czech site of *Drepanocladus sordidus* is also a fishpond adjacent to a peat bog, where one of the largest national populations of Natura´2000 moss, *Hamatocaulis vernicosus* (Mitt.) Hedenäs, occurs (Štechová et al. 2012). *Drepanocladus sordidus* grows in N Poland mainly in soft water lakes (Banaś 2016; Chmara et al. 2018), but in Fennoscandia it also co-occurs with *Calliergon megalophyllum*, even in anthropogenic pools (Uotila 1971).

Both rediscovered moss species should be classed with a hazard category CR – critically endangered in the Czech Republic, according to the criteria for *C. megalophyllum* B2ab (III) D2 and *D. sordidus* A2c A4a B2ab (III) D2.

In our study, accurate and robust morphological species determination is confirmed by genetic homogeneity detected within all analysed KRAM specimens. Representative of *Calliergon megalophyllum* from the Czech Republic in both plastid and nuclear ITS sequence based analyses formed well-supported monophyletic clade together with specimens referred as *C. megalophyllum* from Poland and Sweden. Moreover, the phylogeny position of *C. megalophyllum* is in accordance with Hedenäs et al. (2005) and Hedenäs (2006) investigations. Phylogenetic analysis based on plastid sequences resolved well-supported clade which includes *Dreanocladus sordidus* specimens from the Czech Republic, Poland, Finland, and the United States of America, confirming previous morphological findings. Nevertheless, the described clade, contained also individuals referred to *Pseudocalliergon lycopodioides* and *P. turgescens*. These three bryophyte species (*D. sordidus*, *P. lycopodioides*, *P. turgescens*) shared identical *trn*L-*trn*F, and almost identical (one SNP) *atp*B-*rbc*L plastid sequences. This result, however, is in agreement with the findings of Hedenäs and Rosborg (2008) and Saługa et al. (2018). Phylogenetic analysis based on nuclear ITS sequences also revealed that our original data are homogeneous, but differs from the individual of *D. sordidus* collected in the North America (USA).

In this study, the sequence variation, mainly related to *Pseudocalliergon turgescens*, *P. lycopodioides*, and *Drepanocladus sordidus* specimens, showed poor taxonomic structuring. The most likely explanation for this, regarding plastid data, could be an interspecific hybridisation, a hypothesis often reported in bryophytes (Natcheva and Cronberg 2004, 2007). Meanwhile, ITS phylogenetic analysis may reflect a different evolutionary history for the European and North American representatives. Despite this, the identity of analysed individuals should not raise doubts, since the taxonomic status of these particular specimens was confirmed in the extensive molecular analyses recently published by Hedenäs (2014), Hedenäs and Bisang (2015), and Hedenäs (2017).

In conclusion, our findings, based on morphological and molecular analyses, show that the bryophyte species *Drepanocladus sordidus* and *Calliergon megalophyllum* are present in the Třeboň Basin, Czech Republic, where the latter species form there the southernmost known populations in Europe.

## Supplementary materials

Supplementary material 1 List of 44 sites in the Třeboň Basin, S Bohemia, Czech Republic Data type occurrence Explanation note List of 44 sites in the Třeboň Basin, S Bohemia, Czech Republic, where an extensive search for *Calliergon megalophyllum* and *Drepanocladus sordidus* was conducted in 2017–2018: The following sites were checked: old sand-pit near Spolí-Domanín, Domanínský fishpond near Domanín, pool in a peatbog V Rájích near Spolí, inlet area of Ruda fishpond near Branná, old sand-pit near Ruda fishpond near Branná, Chodec fishpond near Třeboň, Nový u Smitky fishpond near Stará Hlína, humic forest fishpond near Mláka, peatbog at Starý Vdovec fishpond near Stříbřec, peatbogs at Nový Vdovec fishpond near Stříbřec, peatbog near Příbrazský fishpond near Příbraz, peatbog near Výtopa fishpond (W part) near Lutová, humic pool and peatbog near Staré jezero fishpond near Lutová, sand-pit complex Cep I near Suchdol nad Lužnicí, Smržovský fishpond in Smržov, Dvořiště fishpond near Smržov, Potěšil fishpond near Lužnice, Stehlík fishpond near Lomnice nad Lužnicí, fen pools in the extracted fen Karštejn near Val, inlet area of Černiční fishpond near Lužnice, Loužek fishpond near Lužnice, old shallow sand-pit near Ptačí blato fishpond, inlet area of Záblatský fishpond near Záblatí, extracted fen lake near Ponědrážka, pools in the peatbog at Hliníř fishpond near Ponědrážka, peatbog at Švarcenberk fishpond near Ponědrážka, small fishpond in Dunajovická hora near Dunajovice, old sand-pit in Dunajovická hora near Dunajovice, sand-pit near Kramolín, sand-pit near Hluboká u Borovan, small forest fishpond near Jílovice, Žemlička and Horní Rohožný fishponds near Hluboká u Borovan, small humic forest fishpond near Hluboká u Borovan, old extracted sand-pit near the Dračice river near Františkov, old shallow forest sand-pit Bosna near Rapšach, humic Horní Kočvarů fishpond at Velký Londýn near Františkov, old shallow sand-pit at Velký Londýn near Františkov, fen pool in the Dračice river floodplain near Františkov, Skalice, Medenice and Svobodný fishponds near Františkov and humic fishpond Vydýmač in the complex of the Pele peatbog near Chlum u Třeboně.. https://binary.pensoft.net/file/439065This dataset is made available under the Open Database License (http://opendatacommons.org/licenses/odbl/1.0/). The Open Database License (ODbL) is a license agreement intended to allow users to freely share, modify, and use this Dataset while maintaining this same freedom for others, provided that the original source and author(s) are credited.Łukasz Krajewski, Lubomír Adamec, Marta Saługa, Halina Bednarek-Ochyra, Vítězslav Plášek

Supplementary material 2 A comparison of phytosociological releves from world sites of *Calliergon megalophyllum* (and *Drepanocladus sordidus*) from the present and literature data Data type occurrence Explanation note Explanations: S Bohemia, Czech Republic: 1 – Ptačí blato pond, 1st lagoon, 2 – Ptačí blato, fen margin of the 1st pool, 3 - Ptačí blato, 4th lagoon, LA on 27.8.2017, 4 – Branna sand pit; 5 – NE Poland, Suwałki Landscape Park, hollow peat in mire E of Boczniel lake, (+) – present in this mire close to *C. megalophyllum*, but not in the identical habitat, ŁK on 16, 17 and 29 July 2017; 6 –Poland (10 localities; Lisowski 1960, Karczmarz 1971, Ochyra and Tomaszewicz 1982, Ochyra and Szmajda 1983), 7 – NE Netherlands, fen pool (Kooijman et al. 2015), 8 – Sweden (13 sites; Kooijman et al. 2015), 9 – N Germany (Meinunger and Schröder 2007), 10 – S Finland (35 lakes; Karttunen and Toivonen 1995), * – dominant species, + – present species, water depth, pH and EC with median values, (n) – number of records. https://binary.pensoft.net/file/439066This dataset is made available under the Open Database License (http://opendatacommons.org/licenses/odbl/1.0/). The Open Database License (ODbL) is a license agreement intended to allow users to freely share, modify, and use this Dataset while maintaining this same freedom for others, provided that the original source and author(s) are credited.Łukasz Krajewski , Lubomír Adamec, Marta Saługa, Halina Bednarek-Ochyra, Vítězslav Plášek

Supplementary material 3 Sample information and GenBank accession numbers Data type molecular data https://binary.pensoft.net/file/439067This dataset is made available under the Open Database License (http://opendatacommons.org/licenses/odbl/1.0/). The Open Database License (ODbL) is a license agreement intended to allow users to freely share, modify, and use this Dataset while maintaining this same freedom for others, provided that the original source and author(s) are credited.Łukasz Krajewski , Lubomír Adamec, Marta Saługa, Halina Bednarek-Ochyra, Vítězslav Plášek
