# Supplementary material for: Welcome to the Czech Republic again! Rare northern mosses Calliergon megalophyllum and Drepanocladus sordidus (Amblystegiaceae) in South Bohemia in light of their European distribution and habitat preferences
Source: PhytoKeys. 2020 Aug 4;154:111–36. doi: 10.3897/phytokeys.154.51454 (PMC7419341; doi:10.3897/phytokeys.154.51454)
Supplement: Supplementary material 1 — List of 44 sites in the Třeboň Basin, S Bohemia, Czech Republic [file phytokeys-154-111-s001.docx]

**Supplement 1:** List of 44 sites in the Třeboň Basin, S Bohemia, Czech Republic, where an extensive search for *Calliergon megalophyllum* and *Drepanocladus sordidus* was conducted in 2017-2018:

The following sites were checked: old sand-pit near Spolí-Domanín, Domanínský fishpond near Domanín, pool in a peatbog V Rájích near Spolí, inlet area of Ruda fishpond near Branná, old sand-pit near Ruda fishpond near Branná, Chodec fishpond near Třeboň, Nový u Smitky fishpond near Stará Hlína, humic forest fishpond near Mláka, peatbog at Starý Vdovec fishpond near Stříbřec, peatbogs at Nový Vdovec fishpond near Stříbřec, peatbog near Příbrazský fishpond near Příbraz, peatbog near Výtopa fishpond (W part) near Lutová, humic pool and peatbog near Staré jezero fishpond near Lutová, sand-pit complex Cep I near Suchdol nad Lužnicí, Smržovský fishpond in Smržov, Dvořiště fishpond near Smržov, Potěšil fishpond near Lužnice, Stehlík fishpond near Lomnice nad Lužnicí, fen pools in the extracted fen Karštejn near Val, inlet area of Černiční fishpond near Lužnice, Loužek fishpond near Lužnice, old shallow sand-pit near Ptačí blato fishpond, inlet area of Záblatský fishpond near Záblatí, extracted fen lake near Ponědrážka, pools in the peatbog at Hliníř fishpond near Ponědrážka, peatbog at Švarcenberk fishpond near Ponědrážka, small fishpond in Dunajovická hora near Dunajovice, old sand-pit in Dunajovická hora near Dunajovice, sand-pit near Kramolín, sand-pit near Hluboká u Borovan, small forest fishpond near Jílovice, Žemlička and Horní Rohožný fishponds near Hluboká u Borovan, small humic forest fishpond near Hluboká u Borovan, old extracted sand-pit near the Dračice river near Františkov, old shallow forest sand-pit Bosna near Rapšach, humic Horní Kočvarů fishpond at Velký Londýn near Františkov, old shallow sand-pit at Velký Londýn near Františkov, fen pool in the Dračice river floodplain near Františkov, Skalice, Medenice and Svobodný fishponds near Františkov and humic fishpond Vydýmač in the complex of the Pele peatbog near Chlum u Třeboně.
